# Infection pattern, case fatality rate and spread of Lassa virus in Nigeria

**DOI:** 10.1186/s12879-021-05837-x

**Published:** 2021-02-05

**Authors:** Clement Ameh Yaro, Ezekiel Kogi, Kenneth Nnamdi Opara, Gaber El-Saber Batiha, Roua S. Baty, Ashraf Albrakati, Farag M. A. Altalbawy, Innocent Utenwojo Etuh, James Paul Oni

**Affiliations:** 1 Department of Animal and Environmental Biology, University of Uyo, Uyo, Akwa Ibom Nigeria; 2 Department of Zoology, Ahmadu Bello University, Zaria, Nigeria; 3 Department of Pharmacology and Therapeutics, Faculty of Veterinary Medicine, Damanhour University, Damanhour, AlBeheira 22511 Egypt; 4 Department of Biotechnology, College of Science, Taif University, P.O. Box 11099, Taif, 21944 Saudi Arabia; 5 Department of Human Anatomy, College of Medicine, Taif University, P.O. Box 11099, Taif, 21944 Saudi Arabia; 6 National Institute of Laser Enhanced Sciences (NILES), Cairo University, Giza, 12613 Egypt; 7 Department of Animal and Environmental Biology, Kogi State University, Anyigba, Nigeria

**Keywords:** Lassa fever, Lassa virus, Infection pattern, Case fatality, Nigeria

## Abstract

**Background:**

Lassa fever (LF) is a zoonotic infectious disease of public concern in Nigeria. The infection dynamics of the disease is not well elucidated in Nigeria. This study was carried out to describe the pattern of infection, case fatality rate and spread of lassa virus (LASV) from 2017 to 2020.

**Methods:**

Weekly epidemiological data on LF from December, 2016 to September, 2020 were obtained from Nigeria Centre for Disease Control. The number of confirmed cases and deaths were computed according to months and states. Descriptive statistics was performed and case fatality rate was calculated. Distribution and spread maps of LF over the four years period was performed on ArcMap 10.7.

**Results:**

A total of 2787 confirmed cases and 516 deaths were reported in Nigeria from December, 2016 to September, 2020. Increase in number of cases and deaths were observed with 298, 528, 796 and 1165 confirmed cases and 79, 125, 158 and 158 deaths in 2017, 2018, 2019 and 2020 respectively. Over 60% of the cases were reported in two states, Edo and Ondo states. The LF cases spread from 19 states in 2017 to 32 states and Federal Capital Territory (FCT) in 2020. Ondo state (25.39%) had the highest of deaths rate from LF over the four years. Case fatality rate (CFR) of LF was highest in 2017 (26.5%) with CFR of 23.7, 19.6 and 13.4% in 2018, 2019 and 2020 respectively. The peak of infection was in the month of February for the four years. Infections increases at the onset of dry season in November and decline till April when the wet season sets-in.

**Conclusion:**

There is an annual increase in the number of LASV infection across the states in Nigeria. There is need to heighten control strategies through the use of integrated approach, ranging from vector control, health education and early diagnosis.

**Supplementary Information:**

The online version contains supplementary material available at 10.1186/s12879-021-05837-x.

## Background

Lassa fever (LF) also called Lassa hemorrhagic fever is a disease caused by infection with a zoonotic virus called Lassa virus (LASV). LASV is a single-stranded RNA virus of the family Arenaviridae [1] belonging to the genera *Mammarenavirus* [2]. LF was first declared a disease of humans in 1969 within Nigeria [3, 4]. The natural host of LASV is the rodent *Mastomys natalensis*, a common households’ rat in West Africa. *Mastomys erythroleucus* (Guinea multimammate mouse) and *Hylomyscus pamfi* (African wood mouse) are newly reported hosts of the LASV in Nigeria and Guinea Republic [5]. LF is endemic to West Africa especially Nigeria, Liberia and Sierra Leone [1, 6]. Proven cases were also reported in Cote d’Ivoire, Guinea, Central African Republic, Mali, Senegal and Congo [7]. LF is associated with significant morbidity and mortality. The annual incidence of LF in this region is estimated as 100,000 to 300,000 cases with about 5000 deaths and 58 million people at risk [8]. Twenty percent of infected individuals require hospitalization while 80% are asymptomatic infections [9]. The case fatality rate of hospitalized cases ranges from 15 to 20% in Africa [6].

Transmission of the virus to humans occur through direct contact with rat’s excretions such as urine and feces, eating of food and inhalation of contaminated dust containing body secretions of infected rats, as well as eating the rat [6, 7, 10, 11]. Person to person transmission occurs by direct contact with blood or bodily fluids of infected individuals [8, 12]. It can also be transmitted through contact with urine and semen of infected individuals, thereby posing risk for sexual transmission. A recent study reported presence of viral nuclei acid in semen up to 103 days after onset [13]. Infected individuals becomes contagious at the onset of symptom and increases with disease severity [14–16]. Hospitalized patients with LF may pose a significant risk to healthcare workers (HCWs) and to other patients due to its contagious nature [7, 8, 12]. The virus has an incubation period of usually 7–10 days, with a reported range of 3–21 days [6, 12, 14, 16].

Symptoms include fever and malaise, pharyngitis, gastrointestinal complaints, and cough. In later stages bleeding, facial oedema, convulsions, pericardial effusions and coma are commonly observed [7, 12, 17]. Diagnosis is by blood samples which are examined using LASV specific real time reverse-transcriptase polymerase chain reaction (RT-PCR) [18]. The major control strategy is the control of the rodents around dwellings, avoiding of rats consumption and contact [6]. Currently, there are no vaccines against LASV. Although, off-label treatment consider the use of ribavirin [9], an expensive treatment that is effective when administered for the first six days after the onset of symptoms [19].

In Nigeria, the Federal Ministry of Health (FMoH) through the Nigeria Centre for Disease Control (NCDC) established a number of LF case management centres, often called treatment centres to operate in association with specialist teaching hospitals in endemic states. Despite the dangerous nature of this disease, there is scanty information on the seasonal pattern of infections and distribution of LF in Nigeria. This study was carried out to describe the pattern of infection, distribution, spread and case fatality rate of LF in Nigeria over the last four years. The results of this study will provide information for relevant authorities in the design of appropriate strategies and intervention in the control of this virus.

## Methods

### Study area

Nigeria is located on the western coast of Africa. It has a diverse geography with climates ranging from arid to humid equatorial with two distinct seasons (wet season – May to October and dry season – November to April) [20]. Nigeria has a population of over 200 million [21] with a total land area of 923,769 km^2^. The country has 36 states and a Federal Capital Territory (FCT) with 774 Local Government Areas (LGAs) [22].

### LF surveillance in Nigeria

LF surveillance in Nigeria is conducted through the Integrated Disease Surveillance and Response (IDSR) platform. Information on LF flows from the health facilities, through the ward focal persons to the Local Government Area (LGA) Disease Surveillance and Notification Officers (DSNOs), to the State DSNOs, to the State Epidemiologist and then to the NCDC, FMoH. All states in Nigeria including FCT report through the IDSR [22]. Weekly reports on number of confirmed cases and deaths from LF are published in the LF weekly epidemiological reports by the NCDC.

### Source of data

Epidemiological data on LF from December 2016 to September, 2020 were obtained from NCDC weekly reports [23] (Additional file 1).

### Statistical analysis

The data on confirmed cases and confirmed deaths were computed into Microsoft Excel (version 2012; Microsoft Corp., Redmond, USA) according to states and epidemiological weeks. The data obtained were subjected to descriptive statistics. Monthly and annual confirmed cases and deaths of LF was calculated according to states. Case fatality rate (CFR) was calculated using the formula below.

$$ \mathrm{Case}\ \mathrm{Fatality}\ \mathrm{Rate}\ \left(\mathrm{CFR}\right)=\frac{\mathrm{Number}\ \mathrm{of}\ \mathrm{Deaths}}{\mathrm{Number}\ \mathrm{of}\ \mathrm{Case}\mathrm{s}}\ \mathrm{x}\ 100 $$

Analysis was performed using the Statistical Package for Social Sciences (SPSS) software (version 22.0 for windows; SPSS Inc., Chicago, IL, USA). Distribution Map was carried out on ArcMap (version 10.7 for windows; Redland, CA: Environmental Systems Research Institute).

## Results

### Distribution of confirmed cases

An assessment of the weekly reports on LASV infection in Nigeria over the last four years is reported in this study. The result from this study revealed a gradual increase in the number of confirmed cases of LASV. Since the outbreak of LASV in December 2016, Nigeria have reported 2787 confirmed cases of this virus. A total of 298 confirmed cases were observed in 2017 since the outbreak of the disease in December, 2016. In 2018, a total of 528 cases were confirmed, this number almost doubled the number of confirmed cases recorded in 2017 (Table 1). Similar observation were recorded in 2019 and 2020 with confirmed cases of 796 and 1165 respectively.

**Table 1:** Percent Number of Confirmed Cases of LASV in Nigeria

States	Total Number of Confirmed Cases				Total
	2017	2018	2019	2020	
Abia	–	1 (0.19)	1 (0.13)	6 (0.52)	8 (0.29)
Adamawa	–	3 (0.57)	1 (0.13)	7 (0.60)	11 (0.39)
Akwa Ibom	–	–	–	–	–
Anambra	1 (0.34)	6 (1.14)	–	2 (0.17)	9 (0.32)
Bauchi	11 (3.69)	23 (4.36)	55 (6.91)	45 (3.86)	134 (4.81)
Bayelsa	–	–	–	–	–
Benue	–	1 (0.19)	8 (1.01)	9 (0.77)	18 (0.65)
Borno	1 (0.34)	–	–	5 (0.43)	6 (0.22)
Cross River	2 (0.67)	–	1 (0.13)	1 (0.09)	4 (0.14)
Delta	–	7 (1.33)	2 (0.25)	16 (1.37)	25 (0.90)
Ebonyi	5 (1.68)	61 (11.55)	53 (6.66)	82 (7.04)	201 (7.21)
Edo	112 (37.58)	218 (41.29)	289 (36.31)	386 (33.13)	1005 (36.06)
Ekiti	–	2 (0.38)	–	–	2 (0.07)
Enugu	1 (0.34)	1 (0.19)	2 (0.25)	10 (0.86)	14 (0.50)
FCT	–	5 (0.95)	3 (0.38)	3 (0.26)	11 (0.39)
Gombe	1 (0.34)	4 (0.76)	4 (0.50)	11 (0.94)	20 (0.72)
Imo	–	5 (0.95)	1 (0.13)	–	6 (0.22)
Jigawa	–	–	–	–	–
Kaduna	4 (1.34)	1 (0.19)	3 (0.38)	7 (0.60)	15 (0.54)
Kano	7 (2.35)	1 (0.19)	–	7 (0.60)	15 (0.54)
Katsina	–	–	–	6 (0.52)	6 (0.22)
Kebbi	–	–	7 (0.88)	4 (0.34)	11 (0.39)
Kogi	2 (0.67)	7 (1.33)	4 (0.50)	37 (3.18)	50 (1.79)
Kwara	2 (0.67)	–	2 (0.25)	–	4 (0.14)
Lagos	11 (3.69)	1 (0.19)	–	1 (0.09)	13 (0.47)
Nasarawa	15 (5.03)	5 (0.95)	6 (0.75)	9 (0.77)	35 (1.26)
Niger	–	–	–	–	–
Ogun	7 (2.35)	–	–	2 (0.17)	9 (0.32)
Ondo	69 (23.15)	138 (26.14)	273 (34.30)	399 (34.25)	879 (31.54)
Osun	–	2 (0.38)	–	2 (0.17)	4 (0.14)
Oyo	–	–	2 (0.25)	1 (0.09)	3 (0.11)
Plateau	21 (7.05)	15 (2.84)	35 (4.40)	34 (2.92)	105 (3.77)
Rivers	2 (0.67)	1 (0.19)	3 (0.38)	9 (0.77)	15 (0.54)
Sokoto	–	–	–	6 (0.52)	6 (0.22)
Taraba	24 (8.05)	20 (3.79)	40 (5.03)	58 (4.98)	142 (5.10)
Yobe	–	–	–	–	–
Zamfara	–	–	1 (0.13)	–	1 (0.04)
**Total**	**298**	**528**	**796**	**1165**	**2787**

**Fig. 1:**
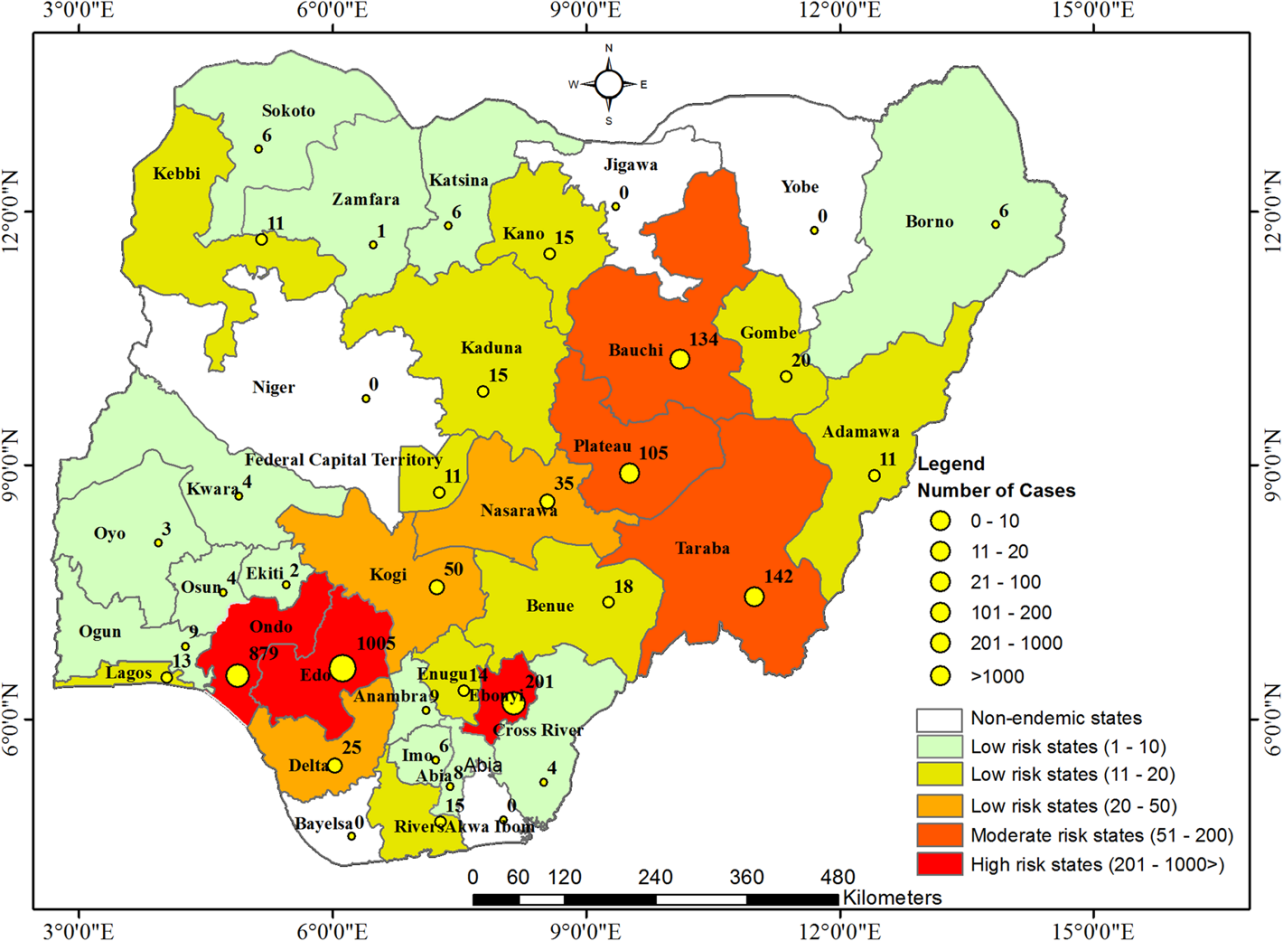
Distribution of Confirmed cases of LASV Infection in Nigeria from 2017 – September, 2020

Majority of the confirmed cases were reported from two states of the federation namely Edo and Ondo states. These two states accounted for 60, 67, 70 and 67% of confirmed cases in 2017, 2018, 2019 and 2020 respectively. Edo and Ondo states reported 37.58% (112 people) and 23.15% (69 people) in 2017, 41.29% (218 people) and 26.14% (138 people) in 2018, 36.31% (289 people) and 34.30% (273 people) in 2019, and 33.13% (386 people) and 34.25% (399 people) in 2020 respectively making these two states the epicenter of LASV in Nigeria (Fig. 1). Increasing number of confirmed cases was observed in these states with year. Edo state is located in the South-South Region and Ondo state in the South-West Region of Nigeria. Consistent cases of LASV were reported from these two states throughout the year. Other states with high cases are Taraba, Plateau, Bauchi and Ebonyi states.

Cumulative confirmed cases for each state over the last four years revealed that Edo state had the highest confirmed cases of 36.06% followed by Ondo state with 31.54%. Other states such as Ebonyi, Taraba, Bauchi and Plateau had appreciable number of confirmed cases of 7.21, 5.10, 4.81 and 3.77% respectively (Table 1). The result revealed increase in number of confirmed cases across many states of the country.

### Spread of LASV

**Fig. 2:**
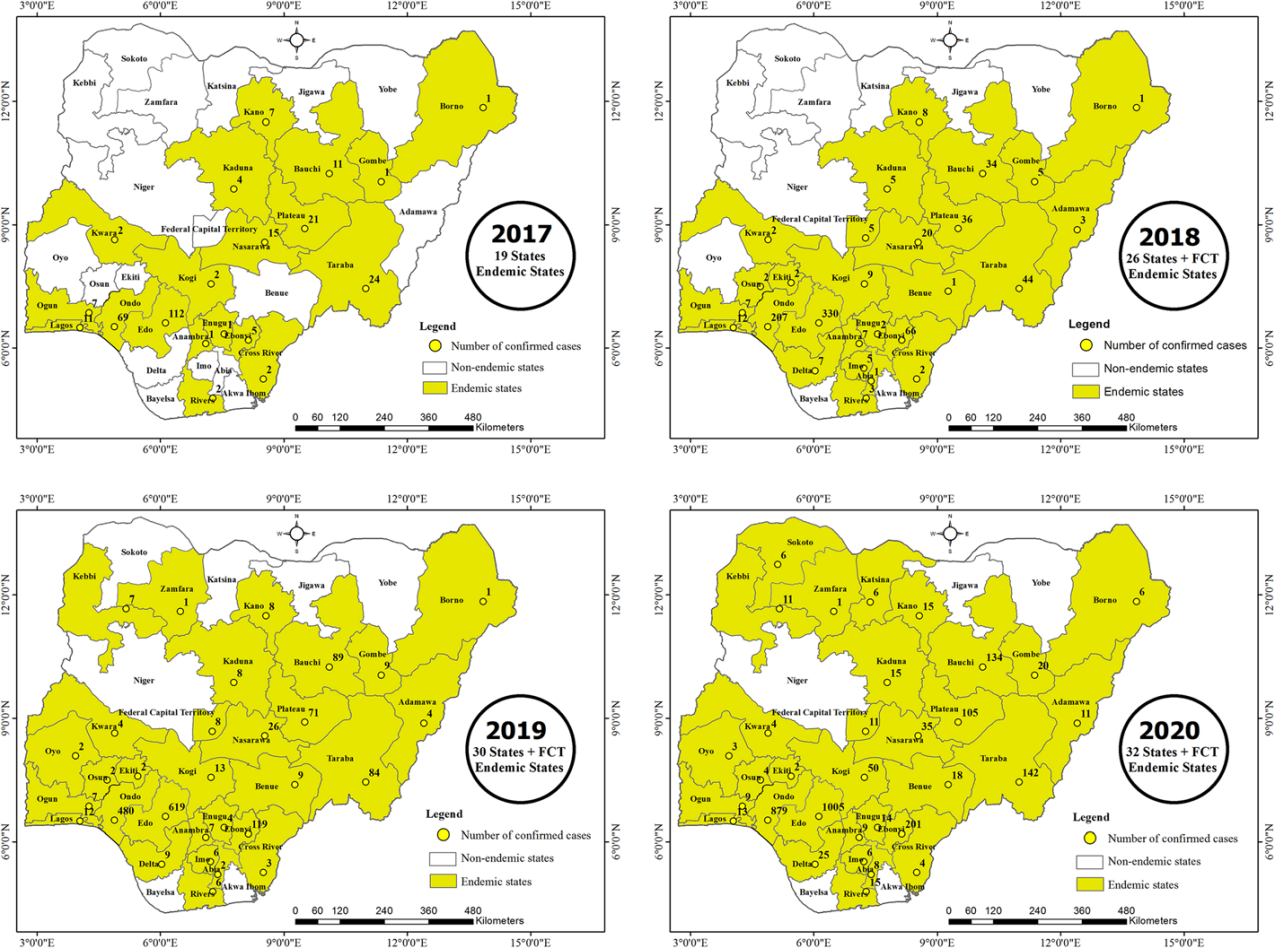
Map of the Geographical Spread of Reported Cases of LF in Nigeria

In 2017, 19 states reported LF cases which further spread to 26 states and the FCT in 2018, 30 states and the FCT in 2019 and to 32 states and the FCT in 2020 (Fig. 2). By September 2020, LASV was reported in 32 states of Nigeria over the last four years (Table 1, Fig. 2). In this study, states previously not endemic for LASV but now have new confirmed cases reported by the NCDC were considered as new geographical spread for the virus.

### Distribution of confirmed death

A total of 516 confirmed death were reported from LF infection in Nigeria over the last four years; 2017–2020. There was an increase in death rate from 79 in 2017 to 156 in 2020.

Death from LF occurred in 15 states in 2017. In 2017, majority of the deaths occurred in Edo state accounting for 17(21.52%) of the death in that year, followed by Taraba and Ondo states with 15.19% (12 people) and 13.92% (11 people) respectively. In 2018, Ondo state had the highest number of confirmed deaths 25(20.0%) from LF, followed by 24(19.20%) in Edo, 23(18.40%) in Ebonyi and 12(9.60%) in Bauchi states. In 2019, Ondo state still maintain the highest death from LF with 45(28.85%) deaths and was closely followed by Edo state with 44(28.21%) while Ebonyi had 17(10.90%) deaths. In 2020, Ondo state still had the highest death rate from LF with 50(32.05%), followed by Edo and Taraba states with 21(13.46%) and 19(12.18%) respectively (Table 2).

**Table 2:** Percent Number of Confirmed Deaths from LF in Nigeria

States	Total Number of Confirmed Death				Total
	2017	2018	2019	2020	
Abia	–	1 (0.80)	1 (0.64)	2 (1.28)	4 (0.78)
Adamawa	–	2 (1.60)	1 (0.64)	1 (0.64)	4 (0.78)
Akwa Ibom	–	–	–	–	–
Anambra	1 (1.27)	1 (0.80)	–	1 (0.64)	3 (0.58)
Bauchi	7 (8.86)	12 (9.60)	8 (5.13)	9 (5.77)	36 (6.98)
Bayelsa	–	–	–	–	–
Benue	–	1 (0.80)	5 (3.21)	3 (1.92)	9 (1.74)
Borno	–	–	–	1 (0.64)	1 (0.19)
Cross River	2 (2.53)	–	1 (0.64)	–	3 (0.58)
Delta	–	2 (1.60)	–	6 (3.85)	8 (1.55)
Ebonyi	1 (1.27)	23 (18.40)	17 (10.90)	13 (8.33)	54 (10.47)
Edo	17 (21.52)	24 (19.20)	44 (28.21)	21 (13.46)	106 (20.54)
Ekiti	–	1 (0.80)	–	–	1 (0.19)
Enugu	–	1 (0.80)	1 (0.64)	2 (1.28)	4 (0.78)
FCT	–	3 (2.40)	2 (1.28)	2 (1.28)	7 (1.36)
Gombe	1 (1.27)	4 (3.20)	1 (0.64)	1 (0.64)	7 (1.36)
Imo	–	–	–	–	–
Jigawa	–	–	–	–	–
Kaduna	2 (2.53)	1 (0.80)	–	2 (1.28)	5 (0.97)
Kano	5 (6.33)	–	–	1 (0.64)	6 (1.16)
Katsina	–	–	–	2 (1.28)	2 (0.39)
Kebbi	–	–	1 (0.64)	1 (0.64)	2 (0.39)
Kogi	1 (1.27)	4 (3.20)	3 (1.92)	5 (3.21)	13 (2.52)
Kwara	–	–	–	–	–
Lagos	3 (3.80)	–	–	–	3 (0.58)
Nasarawa	6 (7.59)	3 (2.40)	4 (2.56)	4 (2.56)	17 (3.29)
Niger	–	–	–	–	–
Ogun	2 (2.53)	–	–	–	2 (0.39)
Ondo	11 (13.92)	25 (20.00)	45 (28.85)	50 (32.05)	131 (25.39)
Osun	–	1 (0.80)	–	–	1 (0.19)
Oyo	–	–	1 (0.64)	1 (0.64)	2 (0.39)
Plateau	8 (10.13)	9 (7.20)	10 (6.41)	5 (3.21)	32 (6.20)
Rivers	–	1 (0.80)	2 (1.28)	3 (1.92)	6 (1.16)
Sokoto	–	–	–	1 (0.64)	1 (0.19)
Taraba	12 (15.19)	6 (4.80)	8 (5.13)	19 (12.18)	45 (8.72)
Yobe	–	–	–	–	–
Zamfara	–	–	1 (0.64)	–	1 (0.19)
**Total**	**79**	**125**	**156**	**156**	**516**

The cumulative death over the four years period revealed that Ondo state had the highest number of deaths from LF with 131(25.39%), followed by Edo state with 106(20.54%), Ebonyi state with 54(10.47%) (Table 2). A total of 30 states in the country have reported deaths from LF over the four period.

### Case fatality rate

The case fatality rate (CFR) from LF in Nigeria was highest at the onset of the outbreak in 2017 with a fatality rate of 26.5%. It decreased little in 2018 to 23.7% and further decrease to 19.6 and 13.4% in 2019 and 2020 respectively (Table 3). The cumulative CFR over the last four years stood at 18.5%. There is fluctuation in the CFR across the states.

**Table 3:** Case Fatality Rate (CFR) of LF in Nigeria

States	Case Fatality Rate				Total
	2017	2018	2019	2020	
Abia		100.0	100.0	33.3	50.0
Adamawa		66.7	100.0	14.3	36.4
Akwa Ibom					
Anambra	100.0	16.7		50.0	33.3
Bauchi	63.6	52.2	14.5	20.0	26.9
Bayelsa					
Benue		100.0	62.5	33.3	50.0
Borno				20.0	16.7
Cross River	100.0		100.0		75.0
Delta		28.6		37.5	32.0
Ebonyi	20.0	37.7	32.1	15.9	26.9
Edo	15.2	11.0	15.2	5.4	10.5
Ekiti		50.0			50.0
Enugu		100.0	50.0	20.0	28.6
FCT		60.0	66.7	66.7	63.6
Gombe	100.0	100.0	25.0	9.1	35.0
Imo					
Jigawa					
Kaduna	50.0	100.0		28.6	33.3
Kano	71.4			14.3	40.0
Katsina				33.3	33.3
Kebbi			14.3	25.0	18.2
Kogi	50.0	57.1	75.0	13.5	26.0
Kwara					
Lagos	27.3				23.1
Nasarawa	40.0	60.0	66.7	44.4	48.6
Niger					
Ogun	28.6				22.2
Ondo	15.9	18.1	16.5	12.5	14.9
Osun		50.0		0.0	25.0
Oyo			50.0	100.0	66.7
Plateau	38.1	60.0	28.6	14.7	30.5
Rivers		100.0	66.7	33.3	40.0
Sokoto				16.7	16.7
Taraba	50.0	30.0	20.0	32.8	31.7
Yobe					
Zamfara			100.0		100.0
**Total**	**26.5**	**23.7**	**19.6**	**13.4**	**18.5**

### Pattern of infections

**Fig. 3:**
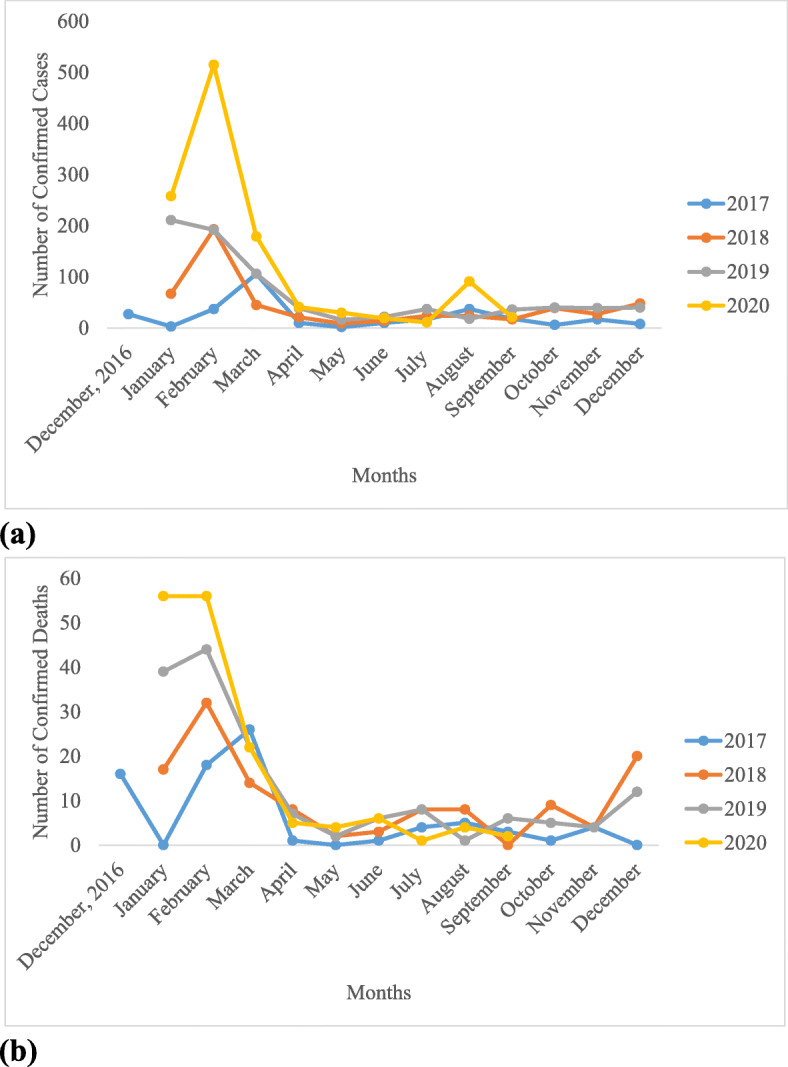
Monthly Distribution of LF (**a**) Confirmed Cases (**b**) Confirmed Deaths

**Fig. 4:**
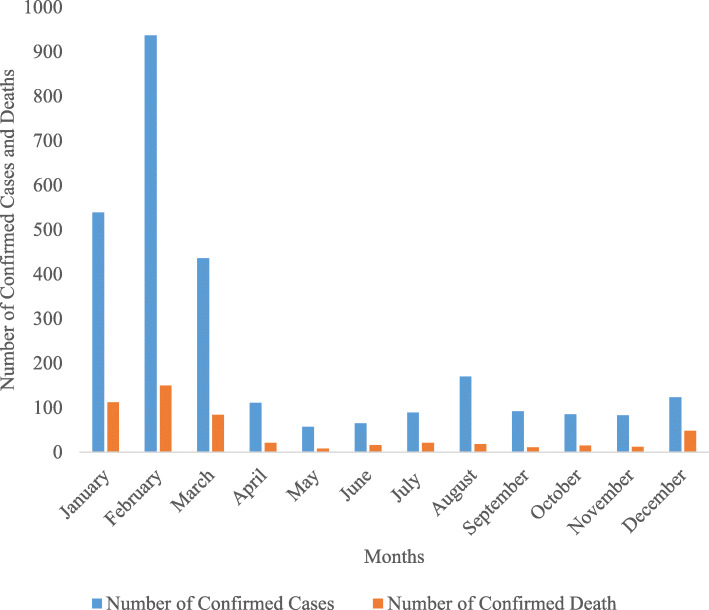
Monthly Number of Confirmed Cases and Deaths of LF Over Four Years Period

The monthly cases of LASV infection over the four years revealed significant difference (*p* < 0.05) across the months of the year. Higher infections and deaths occurred during the dry months than the wet months (Fig. 3). Increase in infections was observed at the onset of the dry season in November and progressively increases in December, January, peaking in February after which it began to decrease until May when the wet season sets in. Highest cases of LASV infection occurred in the month February in the four years of observation. The trend given above was observed throughout the four years (Fig. 3 and Fig. 4).

## Discussion

This study have shown that there is progressive increase in LASV infection in Nigeria, since the outbreak was first reported. The infection exhibited seasonal variability, with the dry season having a significantly higher infection rate than the wet season. The peak infection rate was recorded in February. Similar observation has been reported at Irrua, Edo State [18]. Zhao et al. [24] in their study observed that the major LF epidemic in Nigeria usually occur between November and May during the dry season. A study in Guinea [25] reported that high indoor populations of *M. natalensis* during the dry season than the wet season might have contributed to the higher outbreaks of LASV during the dry season. *M. natalensis* plays a significant role in the rodent to human transmission of the LASV, its high abundance in human dwelling has a direct impact on prevalence. Also, the high reproductive ability of *M. natalensis* is another contributing factor in the spread of the LASV as the rodents can recover its populations within a few months [26]. Human activities such as bush burning which is usually carried out during the dry seasons in the Forest and Guinea Savannah regions of Nigeria where rodents are hunted for meats is another factor favouring the higher prevalence of LASV during the dry season. This activity destroys the rodents’ habitats and thereby encouraging their movement from bushes to human dwellings in search of shelter and food [27–29].

Recently, the LASV has been reported in other rodent hosts other than *M. natalensis*. These new LASV reservoirs are the *Mastomys erythroleucus* (Guinea multimammate mouse) found in Nigeria and Guinea, and *Hylomyscus pamfi* (African wood mouse) in Nigeria [5]. The current presence of LASV in these hosts provides high chances of transmission and resurgence of the virus from time to time. These hosts are found within the same locality with *M. natalensis*, thereby aiding the horizontal transmission of LASV i.e. animal to animal transmission [30]. Some studies reported increase in the territorial habitats of *M. erythroleucus* which is normally a savanna mouse species found in the northern and central part of Nigeria to new localities in southern Nigeria especially in degraded forests [31, 32]. This might explain the current expansion in the geographical spread of LASV across the southern states of the country. In 2017, Olayemi et al. [33] reported the endemicity of LASV in 13 states. In this study, the LASV has reported in 32 states and the FCT as at September, 2020. An evidence that the virus geographical coverage is on the increase. Olayemi et al. [33] further reported that the hosts of LASV i.e. *M. natalensis*, *M. erythroleucus* and *H. pamfi* were distributed across both endemic and non-endemic zones of LF. The presence of these rodents in those communities pose potential risk of spread of LASV.

This study observed that three states in Nigeria; Edo, Ondo and Ebonyi are epicentres for LASV infections. This observation corresponds with earlier reports of NCDC [34] and Usuwa et al. [35]. Series of factors are favouring the increase and spread of LASV infections across Nigeria; the exponential growth of human population, household size, bush burning and urbanisation are some predisposing factors [29]. In Nigeria, the practice of drying agricultural products under the sun especially along roadsides encourages food contamination with urine and faeces of these rodents and hence aiding transmission of LASV [35, 36]. These factors increase human vector contacts thereby putting man at high risk of contracting zoonotic pathogens [37].

## Conclusion

This study revealed yearly increase in the number of infected individuals with LASV. The highest monthly infection from LASV was reported in February for the four years period. Edo and Ondo states still remain the epicenter for the virus, accounting for over 60% of annual cases. The LASV is currently endemic in 32 states and FCT with and annual CFR of 18.5%. Therefore, there is urgent need to declare an emergency against this virus by adopting integrated approach in its control which should involve proper surveillance, health education, proper hosts’ identification, vector control, proper diagnosis, morbidity and adequate training of frontline health professionals.

## Supplementary Information

**Additional file 1.**

## Data Availability

The data sets in this study are available from the corresponding author on reasonable request.

## Abbreviations

LF: Lassa fever
LASV: Lassa virus
HCWs: Healthcare workers
RT-PCR: Reverse-transcriptase polymerase chain reaction
NCDC: Nigeria Centre for Disease
FMoH: Federal Ministry of Health
FCT: Federal Capital Territory
LGAs: Local Government Areas (LGAs)
LGA: Local Government Area
IDSR: Integrated Disease Surveillance and Response
DSNOs: Disease Surveillance and Notification Officers
CFR: Case fatality rate
