# Supplementary material for: Infection pattern, case fatality rate and spread of Lassa virus in Nigeria
Source: BMC Infect Dis. 2021 Feb 5;21:149. doi: 10.1186/s12879-021-05837-x (PMC7863503; doi:10.1186/s12879-021-05837-x)
Supplement: Supplementary file 1 — Additional file 1. [file 12879_2021_5837_MOESM1_ESM.docx]

**Article Type:** Original Research

**Title:** Infection Pattern, Case Fatality Rate and Spread of Lassa Virus in Nigeria.

**Authors:**

*Clement Ameh Yaro^1,2^, Ezekiel Kogi^2^, Kenneth Nnamdi Opara^1^, Gaber El-Saber Batiha^3^, Roua S. Baty^4^, Ashraf Albrakati^5^, Farag M. A. Altalbawy^6^, Innocent Utenwojo Etuh^7^ and James Paul Oni^7^

**Authors Names and Full Addresses:**

***Clement Ameh Yaro, Ph.D**

^1^Department of Animal and Environmental Biology, University of Uyo, Akwa Ibom State, Nigeria

^2^Department of Zoology, Ahmadu Bello University, Zaria, Nigeria.

[yaro.ca@uniuyo.edu.ng](mailto:yaro.ca@uniuyo.edu.ng), +2348064263094. <https://orcid.org/0000-0002-9214-6095>

**Ezekiel Kogi, Ph.D**

^2^Department of Zoology, Ahmadu Bello University, Zaria, Nigeria.

[ekogi.96@gmail.com](mailto:ekogi.96@gmail.com)**,** +2347032341045.

**Kenneth Nnamdi Opara, Ph.D**

^1^Department of Animal and Environmental Biology, University of Uyo, Akwa Ibom State, Nigeria.

[nkopara@yahoo.com](mailto:nkopara@yahoo.com), +238067085205.

**Gaber El-Saber Batiha, Ph.D**

^3^Department of Pharmacology and Therapeutics, Faculty of Veterinary Medicine, Damanhour University, Damanhour 22511, AlBeheira, Egypt.

[gaberbatiha@gmail.com](mailto:gaberbatiha@gmail.com)

**Roua S. Baty**

^4^Department of Biotechnology, College of Science, Taif University, P.O. Box 11099, Taif 21944, Saudi Arabia.

[rsbaty@tu.edu.sa](mailto:rsbaty@tu.edu.sa), <https://orcid.org/0000-0001-5324-0014>

**Ashraf Albrakati**

^5^Department of Human Anatomy, College of Medicine, Taif University, P.O. Box 11099, Taif 21944, Saudi Arabia.

[a.albrakati@tu.edu.sa](mailto:a.albrakati@tu.edu.sa)

**Farag M. A. Altalbawy**

^6^National Institute of Laser Enhanced Sciences (NILES), Cairo University, Giza 12613, Egypt,

[f_altalbawy@yahoo.com](mailto:f_altalbawy@yahoo.com)

**Innocent Utenwojo Etuh, B.Sc**

^7^Department of Animal and Environmental Biology, Kogi State University, Anyigba, Nigeria.

[etuhinnocent@gmail.com](mailto:etuhinnocent@gmail.com)

**James Paul Oni, B.Sc**

^7^Department of Animal and Environmental Biology, Kogi State University, Anyigba, Nigeria.

[onijames4@gmail.com](mailto:onijames4@gmail.com)

**Corresponding Author:**

***Clement Ameh Yaro, Ph.D**

^1^Department of Animal and Environmental Biology, University of Uyo, Akwa Ibom State, Nigeria

^2^Department of Zoology, Ahmadu Bello University, Zaria, Nigeria.

[yaro.ca@uniuyo.edu.ng](mailto:yaro.ca@uniuyo.edu.ng), +2348064263094.

<https://orcid.org/0000-0002-9214-6095>

**SUPPLEMENTARY FILE**

Number of Confirmed cases, 2017

| **States** | **December, 2016** | **January** | **February** | **March** | **April** | **May** | **June** | **July** | **August** | **September** | **October** | **November** | **December** | **Total** |
| --- | --- | --- | --- | --- | --- | --- | --- | --- | --- | --- | --- | --- | --- | --- |
| Abia |  |  |  |  |  |  |  |  |  |  |  |  |  |  |
| Adamawa |  |  |  |  |  |  |  |  |  |  |  |  |  |  |
| Akwa Ibom |  |  |  |  |  |  |  |  |  |  |  |  |  |  |
| Anambra | 0 | 0 | 0 | 0 | 0 | 0 | 1 | 0 | 0 | 0 | 0 | 0 | 0 | **1** |
| Bauchi | 1 | 0 | 2 | 3 | 1 | 0 | 0 | 1 | 0 | 0 | 1 | 2 | 0 | **11** |
| Bayelsa |  |  |  |  |  |  |  |  |  |  |  |  |  |  |
| Benue |  |  |  |  |  |  |  |  |  |  |  |  |  |  |
| Borno | 0 | 0 | 1 | 0 | 0 | 0 | 0 | 0 | 0 | 0 | 0 | 0 | 0 | **1** |
| Cross River | 0 | 0 | 0 | 2 | 0 | 0 | 0 | 0 | 0 | 0 | 0 | 0 | 0 | **2** |
| Delta |  |  |  |  |  |  |  |  |  |  |  |  |  |  |
| Ebonyi | 3 | 0 | 2 | 0 | 0 | 0 | 0 | 0 | 0 | 0 | 0 | 0 | 0 | **5** |
| Edo | 2 | 0 | 5 | 59 | 3 | 2 | 6 | 1 | 12 | 8 | 4 | 9 | 1 | **112** |
| Ekiti |  |  |  |  |  |  |  |  |  |  |  |  |  |  |
| Enugu | 0 | 0 | 0 | 0 | 1 | 0 | 0 | 0 | 0 | 0 | 0 | 0 | 0 | **1** |
| FCT |  |  |  |  |  |  |  |  |  |  |  |  |  |  |
| Gombe | 0 | 0 | 0 | 1 | 0 | 0 | 0 | 0 | 0 | 0 | 0 | 0 | 0 | **1** |
| Imo |  |  |  |  |  |  |  |  |  |  |  |  |  |  |
| Jigawa |  |  |  |  |  |  |  |  |  |  |  |  |  |  |
| Kaduna | 0 | 0 | 1 | 0 | 1 | 0 | 0 | 0 | 1 | 0 | 0 | 1 | 0 | **4** |
| Kano | 0 | 0 | 0 | 5 | 2 | 0 | 0 | 0 | 0 | 0 | 0 | 0 | 0 | **7** |
| Katsina |  |  |  |  |  |  |  |  |  |  |  |  |  |  |
| Kebbi |  |  |  |  |  |  |  |  |  |  |  |  |  |  |
| Kogi | 0 | 0 | 0 | 1 | 0 | 0 | 0 | 0 | 0 | 1 | 0 | 0 | 0 | **2** |
| Kwara | 0 | 0 | 0 | 0 | 0 | 0 | 0 | 0 | 2 | 0 | 0 | 0 | 0 | **2** |
| Lagos | 0 | 0 | 0 | 0 | 0 | 0 | 0 | 0 | 9 | 2 | 0 | 0 | 0 | **11** |
| Nasarawa | 4 | 2 | 2 | 5 | 1 | 0 | 0 | 0 | 0 | 0 | 0 | 0 | 1 | **15** |
| Niger |  |  |  |  |  |  |  |  |  |  |  |  |  |  |
| Ogun | 2 | 0 | 1 | 0 | 0 | 0 | 0 | 0 | 4 | 0 | 0 | 0 | 0 | **7** |
| Ondo | 1 | 1 | 9 | 23 | 1 | 0 | 3 | 7 | 7 | 6 | 1 | 4 | 6 | **69** |
| Osun |  |  |  |  |  |  |  |  |  |  |  |  |  |  |
| Oyo |  |  |  |  |  |  |  |  |  |  |  |  |  |  |
| Plateau | 5 | 0 | 3 | 1 | 0 | 0 | 0 | 8 | 2 | 1 | 0 | 1 | 0 | **21** |
| Rivers | 1 | 0 | 0 | 1 | 0 | 0 | 0 | 0 | 0 | 0 | 0 | 0 | 0 | **2** |
| Sokoto |  |  |  |  |  |  |  |  |  |  |  |  |  |  |
| Taraba | 8 | 0 | 11 | 5 | 0 | 0 | 0 | 0 | 0 | 0 | 0 | 0 | 0 | **24** |
| Yobe |  |  |  |  |  |  |  |  |  |  |  |  |  |  |
| Zamfara |  |  |  |  |  |  |  |  |  |  |  |  |  |  |
| **Total** | **27** | **3** | **37** | **106** | **10** | **2** | **10** | **17** | **37** | **18** | **6** | **17** | **8** | **298** |

Number of Deaths, 2017

| **States** | **December, 2016** | **January** | **February** | **March** | **April** | **May** | **June** | **July** | **August** | **September** | **October** | **November** | **December** | **Total** |
| --- | --- | --- | --- | --- | --- | --- | --- | --- | --- | --- | --- | --- | --- | --- |
| Abia |  |  |  |  |  |  |  |  |  |  |  |  |  |  |
| Adamawa |  |  |  |  |  |  |  |  |  |  |  |  |  |  |
| Akwa Ibom |  |  |  |  |  |  |  |  |  |  |  |  |  |  |
| Anambra | 0 | 0 | 0 | 1 | 0 | 0 | 0 | 0 | 0 | 0 | 0 | 0 | 0 | **1** |
| Bauchi | 1 | 0 | 1 | 1 | 0 | 0 | 0 | 1 | 0 | 0 | 1 | 2 | 0 | **7** |
| Bayelsa |  |  |  |  |  |  |  |  |  |  |  |  |  |  |
| Benue |  |  |  |  |  |  |  |  |  |  |  |  |  |  |
| Borno | 0 | 0 | 0 | 0 | 0 | 0 | 0 | 0 | 0 | 0 | 0 | 0 | 0 | **0** |
| Cross River | 0 | 0 | 0 | 2 | 0 | 0 | 0 | 0 | 0 | 0 | 0 | 0 | 0 | **2** |
| Delta |  |  |  |  |  |  |  |  |  |  |  |  |  |  |
| Ebonyi | 0 | 0 | 1 | 0 | 0 | 0 | 0 | 0 | 0 | 0 | 0 | 0 | 0 | **1** |
| Edo | 4 | 0 | 1 | 10 | 0 | 0 | 0 | 0 | 0 | 1 | 0 | 1 | 0 | **17** |
| Ekiti |  |  |  |  |  |  |  |  |  |  |  |  |  |  |
| Enugu |  |  |  |  |  |  |  |  |  |  |  |  |  |  |
| FCT |  |  |  |  |  |  |  |  |  |  |  |  |  |  |
| Gombe | 0 | 0 | 0 | 1 | 0 | 0 | 0 | 0 | 0 | 0 | 0 | 0 | 0 | **1** |
| Imo |  |  |  |  |  |  |  |  |  |  |  |  |  |  |
| Jigawa |  |  |  |  |  |  |  |  |  |  |  |  |  |  |
| Kaduna | 0 | 0 | 1 | 0 | 0 | 0 | 0 | 0 | 1 | 0 | 0 | 0 | 0 | **2** |
| Kano | 0 | 0 | 0 | 4 | 1 | 0 | 0 | 0 | 0 | 0 | 0 | 0 | 0 | **5** |
| Katsina |  |  |  |  |  |  |  |  |  |  |  |  |  |  |
| Kebbi |  |  |  |  |  |  |  |  |  |  |  |  |  |  |
| Kogi | 0 | 0 | 0 | 0 | 0 | 0 | 0 | 0 | 0 | 1 | 0 | 0 | 0 | **1** |
| Kwara |  |  |  |  |  |  |  |  |  |  |  |  |  |  |
| Lagos | 0 | 0 | 0 | 0 | 0 | 0 | 0 | 0 | 2 | 1 | 0 | 0 | 0 | **3** |
| Nasarawa | 4 | 0 | 0 | 2 | 0 | 0 | 0 | 0 | 0 | 0 | 0 | 0 | 0 | **6** |
| Niger |  |  |  |  |  |  |  |  |  |  |  |  |  |  |
| Ogun | 0 | 0 | 2 | 0 | 0 | 0 | 0 | 0 | 0 | 0 | 0 | 0 | 0 | **2** |
| Ondo | 3 | 0 | 1 | 1 | 0 | 0 | 1 | 2 | 2 | 0 | 0 | 1 | 0 | **11** |
| Osun |  |  |  |  |  |  |  |  |  |  |  |  |  |  |
| Oyo |  |  |  |  |  |  |  |  |  |  |  |  |  |  |
| Plateau | 4 | 0 | 2 | 1 | 0 | 0 | 0 | 1 | 0 | 0 | 0 | 0 | 0 | **8** |
| Rivers | 0 | 0 | 0 | 0 | 0 | 0 | 0 | 0 | 0 | 0 | 0 | 0 | 0 | **0** |
| Sokoto |  |  |  |  |  |  |  |  |  |  |  |  |  |  |
| Taraba | 0 | 0 | 9 | 3 | 0 | 0 | 0 | 0 | 0 | 0 | 0 | 0 | 0 | **12** |
| Yobe |  |  |  |  |  |  |  |  |  |  |  |  |  |  |
| Zamfara |  |  |  |  |  |  |  |  |  |  |  |  |  |  |
| **Total** | **16** | **0** | **18** | **26** | **1** | **0** | **1** | **4** | **5** | **3** | **1** | **4** | **0** | **79** |

Number of confirmed cases, 2018

| **States** | **January** | **February** | **March** | **April** | **May** | **June** | **July** | **August** | **September** | **October** | **November** | **December** | **Total** |
| --- | --- | --- | --- | --- | --- | --- | --- | --- | --- | --- | --- | --- | --- |
| Abia | 0 | 0 | 1 | 0 | 0 | 0 | 0 | 0 | 0 | 0 | 0 | 0 | **1** |
| Adamawa | 0 | 0 | 0 | 1 | 1 | 0 | 0 | 0 | 0 | 0 | 0 | 1 | **3** |
| Akwa Ibom |  |  |  |  |  |  |  |  |  |  |  |  |  |
| Anambra | 1 | 5 | 0 | 0 | 0 | 0 | 0 | 0 | 0 | 0 | 0 | 0 | **6** |
| Bauchi | 1 | 4 | 5 | 0 | 0 | 0 | 0 | 1 | 1 | 0 | 0 | 11 | **23** |
| Bayelsa |  |  |  |  |  |  |  |  |  |  |  |  |  |
| Benue | 1 | 0 | 0 | 0 | 0 | 0 | 0 | 0 | 0 | 0 | 0 | 0 | **1** |
| Borno |  |  |  |  |  |  |  |  |  |  |  |  |  |
| Cross River |  |  |  |  |  |  |  |  |  |  |  |  |  |
| Delta | 1 | 2 | 0 | 0 | 0 | 0 | 0 | 0 | 2 | 1 | 0 | 1 | **7** |
| Ebonyi | 8 | 39 | 3 | 3 | 1 | 0 | 0 | 1 | 0 | 6 | 0 | 0 | **61** |
| Edo | 21 | 78 | 12 | 9 | 3 | 9 | 16 | 18 | 13 | 16 | 9 | 14 | **218** |
| Ekiti | 0 | 1 | 1 | 0 | 0 | 0 | 0 | 0 | 0 | 0 | 0 | 0 | **2** |
| Enugu | 0 | 0 | 0 | 0 | 0 | 0 | 0 | 1 | 0 | 0 | 0 | 0 | **1** |
| FCT | 0 | 2 | 1 | 0 | 0 | 0 | 0 | 0 | 0 | 0 | 0 | 2 | **5** |
| Gombe | 0 | 1 | 1 | 0 | 0 | 0 | 0 | 1 | 0 | 0 | 1 | 0 | **4** |
| Imo | 2 | 2 | 0 | 0 | 0 | 0 | 0 | 0 | 0 | 1 | 0 | 0 | **5** |
| Jigawa |  |  |  |  |  |  |  |  |  |  |  |  |  |
| Kaduna | 0 | 0 | 1 | 0 | 0 | 0 | 0 | 0 | 0 | 0 | 0 | 0 | **1** |
| Kano | 0 | 0 | 0 | 0 | 0 | 0 | 0 | 0 | 0 | 0 | 1 | 0 | **1** |
| Katsina |  |  |  |  |  |  |  |  |  |  |  |  |  |
| Kebbi |  |  |  |  |  |  |  |  |  |  |  |  |  |
| Kogi | 1 | 3 | 1 | 1 | 0 | 0 | 0 | 0 | 0 | 1 | 0 | 0 | **7** |
| Kwara |  |  |  |  |  |  |  |  |  |  |  |  |  |
| Lagos | 1 | 0 | 0 | 0 | 0 | 0 | 0 | 0 | 0 | 0 | 0 | 0 | **1** |
| Nasarawa | 1 | 3 | 0 | 0 | 0 | 0 | 0 | 0 | 0 | 0 | 0 | 1 | **5** |
| Niger |  |  |  |  |  |  |  |  |  |  |  |  |  |
| Ogun |  |  |  |  |  |  |  |  |  |  |  |  |  |
| Ondo | 26 | 45 | 7 | 3 | 3 | 4 | 6 | 2 | 1 | 14 | 15 | 12 | **138** |
| Osun | 0 | 1 | 1 | 0 | 0 | 0 | 0 | 0 | 0 | 0 | 0 | 0 | **2** |
| Oyo |  |  |  |  |  |  |  |  |  |  |  |  |  |
| Plateau | 0 | 4 | 3 | 1 | 0 | 1 | 1 | 0 | 0 | 0 | 1 | 4 | **15** |
| Rivers | 0 | 1 | 0 | 0 | 0 | 0 | 0 | 0 | 0 | 0 | 0 | 0 | **1** |
| Sokoto |  |  |  |  |  |  |  |  |  |  |  |  |  |
| Taraba | 3 | 2 | 8 | 3 | 1 | 0 | 1 | 0 | 0 | 0 | 0 | 2 | **20** |
| Yobe |  |  |  |  |  |  |  |  |  |  |  |  |  |
| Zamfara |  |  |  |  |  |  |  |  |  |  |  |  |  |
| **Total** | **67** | **193** | **45** | **21** | **9** | **14** | **24** | **24** | **17** | **39** | **27** | **48** | **528** |

Number of Deaths, 2018

| **States** | **January** | **February** | **March** | **April** | **May** | **June** | **July** | **August** | **September** | **October** | **November** | **December** | **Total** |
| --- | --- | --- | --- | --- | --- | --- | --- | --- | --- | --- | --- | --- | --- |
| Abia | 0 | 0 | 1 | 0 | **0** | 0 | 0 | 0 | 0 | 0 | 0 | 0 | **1** |
| Adamawa | 0 | 0 | 0 | 1 | **0** | 0 | 0 | 0 | 0 | 0 | 0 | 1 | **2** |
| Akwa Ibom |  |  |  |  |  |  |  |  |  |  |  |  |  |
| Anambra | 0 | 1 | 0 | 0 | **0** | 0 | 0 | 0 | 0 | 0 | 0 | 0 | **1** |
| Bauchi | 0 | 4 | 0 | 0 | **0** | 0 | 0 | 1 | 0 | 0 | 0 | 7 | **12** |
| Bayelsa |  |  |  |  |  |  |  |  |  |  |  |  |  |
| Benue | 1 | 0 | 0 | 0 | **0** | 0 | 0 | 0 | 0 | 0 | 0 | 0 | **1** |
| Borno |  |  |  |  |  |  |  |  |  |  |  |  |  |
| Cross River |  |  |  |  |  |  |  |  |  |  |  |  |  |
| Delta | 0 | 1 | 0 | 0 | **0** | 0 | 0 | 0 | 0 | 0 | 0 | 1 | **2** |
| Ebonyi | 13 | 3 | 2 | 2 | **0** | 0 | 0 | 1 | 0 | 2 | 0 | 0 | **23** |
| Edo | 0 | 5 | 3 | 1 | **0** | 2 | 4 | 4 | 0 | 4 | 1 | 0 | **24** |
| Ekiti | 0 | 1 | 0 | 0 | **0** | 0 | 0 | 0 | 0 | 0 | 0 | 0 | **1** |
| Enugu | 0 | 0 | 0 | 0 | **0** | 0 | 0 | 1 | 0 | 0 | 0 | 0 | **1** |
| FCT | 0 | 1 | 1 | 0 | **0** | 0 | 0 | 0 | 0 | 0 | 0 | 1 | **3** |
| Gombe | 0 | 1 | 1 | 0 | **0** | 0 | 0 | 1 | 0 | 0 | 1 | 0 | **4** |
| Imo |  |  |  |  |  |  |  |  |  |  |  |  |  |
| Jigawa |  |  |  |  |  |  |  |  |  |  |  |  |  |
| Kaduna | 0 | 0 | 1 | 0 | **0** | 0 | 0 | 0 | 0 | 0 | 0 | 0 | **1** |
| Kano |  |  |  |  |  |  |  |  |  |  |  |  |  |
| Katsina |  |  |  |  |  |  |  |  |  |  |  |  |  |
| Kebbi |  |  |  |  |  |  |  |  |  |  |  |  |  |
| Kogi | 2 | 0 | 0 | 2 | **0** | 0 | 0 | 0 | 0 | 0 | 0 | 0 | **4** |
| Kwara |  |  |  |  |  |  |  |  |  |  |  |  |  |
| Lagos |  |  |  |  |  |  |  |  |  |  |  |  |  |
| Nasarawa | 1 | 1 | 0 | 0 | **0** | 0 | 0 | 0 | 0 | 0 | 0 | 1 | **3** |
| Niger |  |  |  |  |  |  |  |  |  |  |  |  |  |
| Ogun |  |  |  |  |  |  |  |  |  |  |  |  |  |
| Ondo | 0 | 10 | 0 | 1 | **1** | 0 | 3 | 0 | 0 | 3 | 1 | 6 | **25** |
| Osun | 0 | 1 | 0 | 0 | **0** | 0 | 0 | 0 | 0 | 0 | 0 | 0 | **1** |
| Oyo |  |  |  |  |  |  |  |  |  |  |  |  |  |
| Plateau | 0 | 2 | 2 | 0 | **0** | 1 | 1 | 0 | 0 | 0 | 1 | 2 | **9** |
| Rivers | 0 | 1 | 0 | 0 | **0** | 0 | 0 | 0 | 0 | 0 | 0 | 0 | **1** |
| Sokoto |  |  |  |  |  |  |  |  |  |  |  |  |  |
| Taraba | 0 | 0 | 3 | 1 | **1** | 0 | 0 | 0 | 0 | 0 | 0 | 1 | **6** |
| Yobe |  |  |  |  |  |  |  |  |  |  |  |  |  |
| Zamfara |  |  |  |  |  |  |  |  |  |  |  |  |  |
| **Total** | **17** | **32** | **14** | **8** | **2** | **3** | **8** | **8** | **0** | **9** | **4** | **20** | **125** |

Number of confirmed cases, 2019

| **States** | **January** | **February** | **March** | **April** | **May** | **June** | **July** | **August** | **September** | **October** | **November** | **December** | **Total** |
| --- | --- | --- | --- | --- | --- | --- | --- | --- | --- | --- | --- | --- | --- |
| Abia | 0 | 0 | 0 | 0 | 0 | 0 | 0 | 0 | 0 | 0 | 1 | 0 | **1** |
| Adamawa | 1 | 0 | 0 | 0 | 0 | 0 | 0 | 0 | 0 | 0 | 0 | 0 | **1** |
| Akwa Ibom |  |  |  |  |  |  |  |  |  |  |  |  |  |
| Anambra |  |  |  |  |  |  |  |  |  |  |  |  |  |
| Bauchi | 16 | 15 | 9 | 2 | 0 | 0 | 3 | 2 | 0 | 2 | 2 | 4 | **55** |
| Bayelsa |  |  |  |  |  |  |  |  |  |  |  |  |  |
| Benue | 2 | 2 | 1 | 0 | 0 | 1 | 0 | 0 | 1 | 1 | 0 | 0 | **8** |
| Borno |  |  |  |  |  |  |  |  |  |  |  |  |  |
| Cross River | 0 | 1 | 0 | 0 | 0 | 0 | 0 | 0 | 0 | 0 | 0 | 0 | **1** |
| Delta | 0 | 1 | 1 | 0 | 0 | 0 | 0 | 0 | 0 | 0 | 0 | 0 | **2** |
| Ebonyi | 13 | 14 | 13 | 1 | 3 | 0 | 3 | 0 | 1 | 0 | 1 | 4 | **53** |
| Edo | 76 | 67 | 41 | 8 | 8 | 11 | 15 | 11 | 16 | 20 | 12 | 4 | **289** |
| Ekiti |  |  |  |  |  |  |  |  |  |  |  |  |  |
| Enugu | 1 | 0 | 1 | 0 | 0 | 0 | 0 | 0 | 0 | 0 | 0 | 0 | **2** |
| FCT | 3 | 0 | 0 | 0 | 0 | 0 | 0 | 0 | 0 | 0 | 0 | 0 | **3** |
| Gombe | 1 | 2 | 0 | 0 | 0 | 0 | 0 | 0 | 0 | 0 | 0 | 1 | **4** |
| Imo | 0 | 0 | 1 | 0 | 0 | 0 | 0 | 0 | 0 | 0 | 0 | 0 | **1** |
| Jigawa |  |  |  |  |  |  |  |  |  |  |  |  |  |
| Kaduna | 1 | 2 | 0 | 0 | 0 | 0 | 0 | 0 | 0 | 0 | 0 | 0 | **3** |
| Kano |  |  |  |  |  |  |  |  |  |  |  |  |  |
| Katsina |  |  |  |  |  |  |  |  |  |  |  |  |  |
| Kebbi | 0 | 2 | 1 | 3 | 0 | 0 | 1 | 0 | 0 | 0 | 0 | 0 | **7** |
| Kogi | 1 | 2 | 0 | 0 | 0 | 1 | 0 | 0 | 0 | 0 | 0 | 0 | **4** |
| Kwara | 1 | 1 | 0 | 0 | 0 | 0 | 0 | 0 | 0 | 0 | 0 | 0 | **2** |
| Lagos |  |  |  |  |  |  |  |  |  |  |  |  |  |
| Nasarawa | 1 | 3 | 2 | 0 | 0 | 0 | 0 | 0 | 0 | 0 | 0 | 0 | **6** |
| Niger |  |  |  |  |  |  |  |  |  |  |  |  |  |
| Ogun |  |  |  |  |  |  |  |  |  |  |  |  |  |
| Ondo | 66 | 50 | 22 | 18 | 3 | 9 | 15 | 5 | 18 | 17 | 23 | 27 | **273** |
| Osun |  |  |  |  |  |  |  |  |  |  |  |  |  |
| Oyo | 0 | 2 | 0 | 0 | 0 | 0 | 0 | 0 | 0 | 0 | 0 | 0 | **2** |
| Plateau | 17 | 10 | 5 | 3 | 0 | 0 | 0 | 0 | 0 | 0 | 0 | 0 | **35** |
| Rivers | 1 | 2 | 0 | 0 | 0 | 0 | 0 | 0 | 0 | 0 | 0 | 0 | **3** |
| Sokoto |  |  |  |  |  |  |  |  |  |  |  |  |  |
| Taraba | 10 | 16 | 9 | 4 | 1 | 0 | 0 | 0 | 0 | 0 | 0 | 0 | **40** |
| Yobe |  |  |  |  |  |  |  |  |  |  |  |  |  |
| Zamfara | 0 | 0 | 0 | 0 | 1 | 0 | 0 | 0 | 0 | 0 | 0 | 0 | **1** |
| **Total** | **211** | **192** | **106** | **39** | **16** | **22** | **37** | **18** | **36** | **40** | **39** | **40** | **796** |

Number of Deaths, 2019

| **States** | **January** | **February** | **March** | **April** | **May** | **June** | **July** | **August** | **September** | **October** | **November** | **December** | **Total** |
| --- | --- | --- | --- | --- | --- | --- | --- | --- | --- | --- | --- | --- | --- |
| Abia | 0 | 0 | 0 | 0 | 0 | 0 | 0 | 0 | 0 | 0 | 1 | 0 | **1** |
| Adamawa | 1 | 0 | 0 | 0 | 0 | 0 | 0 | 0 | 0 | 0 | 0 | 0 | **1** |
| Akwa Ibom |  |  |  |  |  |  |  |  |  |  |  |  |  |
| Anambra |  |  |  |  |  |  |  |  |  |  |  |  |  |
| Bauchi | 1 | 3 | 3 | 0 | 0 | 0 | 0 | 0 | 0 | 0 | 0 | 1 | **8** |
| Bayelsa |  |  |  |  |  |  |  |  |  |  |  |  |  |
| Benue | 1 | 0 | 1 | 0 | 0 | 1 | 0 | 0 | 1 | 1 | 0 | 0 | **5** |
| Borno |  |  |  |  |  |  |  |  |  |  |  |  |  |
| Cross River | 0 | 1 | 0 | 0 | 0 | 0 | 0 | 0 | 0 | 0 | 0 | 0 | **1** |
| Delta |  |  |  |  |  |  |  |  |  |  |  |  |  |
| Ebonyi | 2 | 8 | 4 | 0 | 0 | 0 | 3 | 0 | 0 | 0 | 0 | 0 | **17** |
| Edo | 10 | 10 | 7 | 1 | 1 | 1 | 4 | 0 | 4 | 3 | 1 | 2 | **44** |
| Ekiti |  |  |  |  |  |  |  |  |  |  |  |  |  |
| Enugu | 0 | 1 | 0 | 0 | 0 | 0 | 0 | 0 | 0 | 0 | 0 | 0 | **1** |
| FCT | 2 | 0 | 0 | 0 | 0 | 0 | 0 | 0 | 0 | 0 | 0 | 0 | **2** |
| Gombe | 0 | 0 | 0 | 0 | 0 | 0 | 0 | 0 | 0 | 0 | 0 | 1 | **1** |
| Imo |  |  |  |  |  |  |  |  |  |  |  |  |  |
| Jigawa |  |  |  |  |  |  |  |  |  |  |  |  |  |
| Kaduna |  |  |  |  |  |  |  |  |  |  |  |  |  |
| Kano |  |  |  |  |  |  |  |  |  |  |  |  |  |
| Katsina |  |  |  |  |  |  |  |  |  |  |  |  |  |
| Kebbi | 0 | 0 | 0 | 1 | 0 | 0 | 0 | 0 | 0 | 0 | 0 | 0 | **1** |
| Kogi | 0 | 2 | 0 | 0 | 0 | 1 | 0 | 0 | 0 | 0 | 0 | 0 | **3** |
| Kwara |  |  |  |  |  |  |  |  |  |  |  |  |  |
| Lagos |  |  |  |  |  |  |  |  |  |  |  |  |  |
| Nasarawa | 1 | 2 | 1 | 0 | 0 | 0 | 0 | 0 | 0 | 0 | 0 | 0 | **4** |
| Niger |  |  |  |  |  |  |  |  |  |  |  |  |  |
| Ogun |  |  |  |  |  |  |  |  |  |  |  |  |  |
| Ondo | 10 | 9 | 5 | 4 | 0 | 3 | 1 | 1 | 1 | 1 | 2 | 8 | **45** |
| Osun |  |  |  |  |  |  |  |  |  |  |  |  |  |
| Oyo | 0 | 1 | 0 | 0 | 0 | 0 | 0 | 0 | 0 | 0 | 0 | 0 | **1** |
| Plateau | 6 | 4 | 0 | 0 | 0 | 0 | 0 | 0 | 0 | 0 | 0 | 0 | **10** |
| Rivers | 1 | 1 | 0 | 0 | 0 | 0 | 0 | 0 | 0 | 0 | 0 | 0 | **2** |
| Sokoto |  |  |  |  |  |  |  |  |  |  |  |  |  |
| Taraba | 4 | 2 | 1 | 1 | 0 | 0 | 0 | 0 | 0 | 0 | 0 | 0 | **8** |
| Yobe |  |  |  |  |  |  |  |  |  |  |  |  |  |
| Zamfara | 0 | 0 | 0 | 0 | 1 | 0 | 0 | 0 | 0 | 0 | 0 | 0 | **1** |
| **Total** | **39** | **44** | **22** | **7** | **2** | **6** | **8** | **1** | **6** | **5** | **4** | **12** | **156** |

Number of confirmed cases, 2020

| **States** | **January** | **February** | **March** | **April** | **May** | **June** | **July** | **August** | **September** | **October** | **November** | **December** | **Total** |
| --- | --- | --- | --- | --- | --- | --- | --- | --- | --- | --- | --- | --- | --- |
| Abia | 2 | 2 | 0 | 0 | 0 | 0 | 0 | 2 | 0 |  |  |  | **6** |
| Adamawa | 1 | 2 | 4 | 0 | 0 | 0 | 0 | 0 | 0 |  |  |  | **7** |
| Akwa Ibom |  |  |  |  |  |  |  |  |  |  |  |  |  |
| Anambra | 0 | 1 | 1 | 0 | 0 | 0 | 0 | 0 | 0 |  |  |  | **2** |
| Bauchi | 7 | 26 | 9 | 1 | 1 | 0 | 0 | 1 | 0 |  |  |  | **45** |
| Bayelsa |  |  |  |  |  |  |  |  |  |  |  |  |  |
| Benue | 1 | 6 | 1 | 0 | 1 | 0 | 0 | 0 | 0 |  |  |  | **9** |
| Borno | 3 | 1 | 0 | 0 | 0 | 0 | 0 | 1 | 0 |  |  |  | **5** |
| Cross River | 0 | 0 | 0 | 0 | 0 | 0 | 0 | 1 | 0 |  |  |  | **1** |
| Delta | 5 | 8 | 3 | 0 | 0 | 0 | 0 | 0 | 0 |  |  |  | **16** |
| Ebonyi | 15 | 38 | 18 | 1 | 3 | 0 | 0 | 7 | 0 |  |  |  | **82** |
| Edo | 92 | 173 | 47 | 12 | 9 | 5 | 2 | 38 | 8 |  |  |  | **386** |
| Ekiti |  |  |  |  |  |  |  |  |  |  |  |  |  |
| Enugu | 2 | 5 | 3 | 0 | 0 | 0 | 0 | 0 | 0 |  |  |  | **10** |
| FCT | 0 | 1 | 2 | 0 | 0 | 0 | 0 | 0 | 0 |  |  |  | **3** |
| Gombe | 0 | 4 | 2 | 3 | 0 | 0 | 0 | 2 | 0 |  |  |  | **11** |
| Imo |  |  |  |  |  |  |  |  |  |  |  |  |  |
| Jigawa |  |  |  |  |  |  |  |  |  |  |  |  |  |
| Kaduna | 1 | 5 | 1 | 0 | 0 | 0 | 0 | 0 | 0 |  |  |  | **7** |
| Kano | 3 | 3 | 0 | 0 | 0 | 0 | 0 | 1 | 0 |  |  |  | **7** |
| Katsina | 0 | 5 | 1 | 0 | 0 | 0 | 0 | 0 | 0 |  |  |  | **6** |
| Kebbi | 0 | 3 | 1 | 0 | 0 | 0 | 0 | 0 | 0 |  |  |  | **4** |
| Kogi | 4 | 21 | 7 | 3 | 1 | 0 | 0 | 0 | 1 |  |  |  | **37** |
| Kwara |  |  |  |  |  |  |  |  |  |  |  |  |  |
| Lagos | 0 | 1 | 0 | 0 | 0 | 0 | 0 | 0 | 0 |  |  |  | **1** |
| Nasarawa | 1 | 5 | 1 | 2 | 0 | 0 | 0 | 0 | 0 |  |  |  | **9** |
| Niger |  |  |  |  |  |  |  |  |  |  |  |  |  |
| Ogun | 2 | 0 | 0 | 0 | 0 | 0 | 0 | 0 | 0 |  |  |  | **2** |
| Ondo | 97 | 144 | 59 | 15 | 14 | 12 | 9 | 37 | 12 |  |  |  | **399** |
| Osun | 2 | 0 | 0 | 0 | 0 | 0 | 0 | 0 | 0 |  |  |  | **2** |
| Oyo | 0 | 1 | 0 | 0 | 0 | 0 | 0 | 0 | 0 |  |  |  | **1** |
| Plateau | 7 | 17 | 5 | 2 | 1 | 1 | 0 | 1 | 0 |  |  |  | **34** |
| Rivers | 3 | 6 | 0 | 0 | 0 | 0 | 0 | 0 | 0 |  |  |  | **9** |
| Sokoto | 0 | 4 | 1 | 1 | 0 | 0 | 0 | 0 | 0 |  |  |  | **6** |
| Taraba | 10 | 33 | 13 | 1 | 0 | 1 | 0 | 0 | 0 |  |  |  | **58** |
| Yobe |  |  |  |  |  |  |  |  |  |  |  |  |  |
| Zamfara |  |  |  |  |  |  |  |  |  |  |  |  |  |
| **Total** | **258** | **515** | **179** | **41** | **30** | **19** | **11** | **91** | **21** |  |  |  | **1165** |

Number of Deaths, 2020

| **States** | **January** | **February** | **March** | **April** | **May** | **June** | **July** | **August** | **September** | **October** | **November** | **December** | **Total** |
| --- | --- | --- | --- | --- | --- | --- | --- | --- | --- | --- | --- | --- | --- |
| Abia | 2 | 0 | 0 | 0 | 0 | 0 | 0 | 0 | 0 |  |  |  | **2** |
| Adamawa | 1 | 0 | 0 | 0 | 0 | 0 | 0 | 0 | 0 |  |  |  | **1** |
| Akwa Ibom |  |  |  |  |  |  |  |  |  |  |  |  |  |
| Anambra | 0 | 0 | 1 | 0 | 0 | 0 | 0 | 0 | 0 |  |  |  | **1** |
| Bauchi | 2 | 6 | 1 | 0 | 0 | 0 | 0 | 0 | 0 |  |  |  | **9** |
| Bayelsa |  |  |  |  |  |  |  |  |  |  |  |  |  |
| Benue | 0 | 1 | 0 | 1 | 1 | 0 | 0 | 0 | 0 |  |  |  | **3** |
| Borno | 1 | 0 | 0 | 0 | 0 | 0 | 0 | 0 | 0 |  |  |  | **1** |
| Cross River |  |  |  |  |  |  |  |  |  |  |  |  |  |
| Delta | 4 | 1 | 1 | 0 | 0 | 0 | 0 | 0 | 0 |  |  |  | **6** |
| Ebonyi | 6 | 4 | 2 | 1 | 0 | 0 | 0 | 0 | 0 |  |  |  | **13** |
| Edo | 6 | 10 | 5 | 0 | 0 | 0 | 0 | 0 | 0 |  |  |  | **21** |
| Ekiti |  |  |  |  |  |  |  |  |  |  |  |  |  |
| Enugu | 1 | 0 | 1 | 0 | 0 | 0 | 0 | 0 | 0 |  |  |  | **2** |
| FCT | 0 | 0 | 2 | 0 | 0 | 0 | 0 | 0 | 0 |  |  |  | **2** |
| Gombe | 0 | 1 | 0 | 0 | 0 | 0 | 0 | 0 | 0 |  |  |  | **1** |
| Imo |  |  |  |  |  |  |  |  |  |  |  |  |  |
| Jigawa |  |  |  |  |  |  |  |  |  |  |  |  |  |
| Kaduna | 1 | 1 | 0 | 0 | 0 | 0 | 0 | 0 | 0 |  |  |  | **2** |
| Kano | 1 | 0 | 0 | 0 | 0 | 0 | 0 | 0 | 0 |  |  |  | **1** |
| Katsina | 0 | 2 | 0 | 0 | 0 | 0 | 0 | 0 | 0 |  |  |  | **2** |
| Kebbi | 0 | 1 | 0 | 0 | 0 | 0 | 0 | 0 | 0 |  |  |  | **1** |
| Kogi | 3 | 2 | 0 | 0 | 0 | 0 | 0 | 0 | 0 |  |  |  | **5** |
| Kwara |  |  |  |  |  |  |  |  |  |  |  |  |  |
| Lagos |  |  |  |  |  |  |  |  |  |  |  |  |  |
| Nasarawa | 1 | 2 | 0 | 1 | 0 | 0 | 0 | 0 | 0 |  |  |  | **4** |
| Niger |  |  |  |  |  |  |  |  |  |  |  |  |  |
| Ogun |  |  |  |  |  |  |  |  |  |  |  |  |  |
| Ondo | 16 | 11 | 7 | 2 | 3 | 4 | 1 | 4 | 2 |  |  |  | **50** |
| Osun |  |  |  |  |  |  |  |  |  |  |  |  |  |
| Oyo | 1 | 0 | 0 | 0 | 0 | 0 | 0 | 0 | 0 |  |  |  | **1** |
| Plateau | 0 | 3 | 1 | 0 | 0 | 1 | 0 | 0 | 0 |  |  |  | **5** |
| Rivers | 3 | 0 | 0 | 0 | 0 | 0 | 0 | 0 | 0 |  |  |  | **3** |
| Sokoto | 0 | 1 | 0 | 0 | 0 | 0 | 0 | 0 | 0 |  |  |  | **1** |
| Taraba | 7 | 10 | 1 | 0 | 0 | 1 | 0 | 0 | 0 |  |  |  | **19** |
| Yobe |  |  |  |  |  |  |  |  |  |  |  |  |  |
| Zamfara |  |  |  |  |  |  |  |  |  |  |  |  |  |
| **Total** | **56** | **56** | **22** | **5** | **4** | **6** | **1** | **4** | **2** |  |  |  | **156** |

Number of Confirmed Cases from 2017 to 2020 According to States

| **States** | **Total Number of Confirmed Cases** | | | | **Total** |
| --- | --- | --- | --- | --- | --- |
|  | **2017** | **2018** | **2019** | **2020** |  |
| Abia |  | **1** | **1** | **6** | **8** |
| Adamawa |  | **3** | **1** | **7** | **11** |
| Akwa Ibom |  |  |  |  |  |
| Anambra | **1** | **6** |  | **2** | **9** |
| Bauchi | **11** | **23** | **55** | **45** | **134** |
| Bayelsa |  |  |  |  |  |
| Benue |  | **1** | **8** | **9** | **18** |
| Borno | **1** |  |  | **5** | **6** |
| Cross River | **2** |  | **1** | **1** | **4** |
| Delta |  | **7** | **2** | **16** | **25** |
| Ebonyi | **5** | **61** | **53** | **82** | **201** |
| Edo | **112** | **218** | **289** | **386** | **1005** |
| Ekiti |  | **2** |  |  | **2** |
| Enugu | **1** | **1** | **2** | **10** | **14** |
| FCT |  | **5** | **3** | **3** | **11** |
| Gombe | **1** | **4** | **4** | **11** | **20** |
| Imo |  | **5** | **1** |  | **6** |
| Jigawa |  |  |  |  |  |
| Kaduna | **4** | **1** | **3** | **7** | **15** |
| Kano | **7** | **1** |  | **7** | **15** |
| Katsina |  |  |  | **6** | **6** |
| Kebbi |  |  | **7** | **4** | **11** |
| Kogi | **2** | **7** | **4** | **37** | **50** |
| Kwara | **2** |  | **2** |  | **4** |
| Lagos | **11** | **1** |  | **1** | **13** |
| Nasarawa | **15** | **5** | **6** | **9** | **35** |
| Niger |  |  |  |  |  |
| Ogun | **7** |  |  | **2** | **9** |
| Ondo | **69** | **138** | **273** | **399** | **879** |
| Osun |  | **2** |  | **2** | **4** |
| Oyo |  |  | **2** | **1** | **3** |
| Plateau | **21** | **15** | **35** | **34** | **105** |
| Rivers | **2** | **1** | **3** | **9** | **15** |
| Sokoto |  |  |  | **6** | **6** |
| Taraba | **24** | **20** | **40** | **58** | **142** |
| Yobe |  |  |  |  |  |
| Zamfara |  |  | **1** |  | **1** |
| **Total** | **298** | **528** | **796** | **1165** | **2787** |

Number of Deaths from 2017 to 2020 According to States

| **States** | **Total Number of Confirmed Death** | | | | **Total** |
| --- | --- | --- | --- | --- | --- |
|  | **2017** | **2018** | **2019** | **2020** |  |
| Abia |  | **1** | **1** | **2** | **4** |
| Adamawa |  | **2** | **1** | **1** | **4** |
| Akwa Ibom |  |  |  |  | **0** |
| Anambra | **1** | **1** |  | **1** | **3** |
| Bauchi | **7** | **12** | **8** | **9** | **36** |
| Bayelsa |  |  |  |  | **0** |
| Benue |  | **1** | **5** | **3** | **9** |
| Borno |  |  |  | **1** | **1** |
| Cross River | **2** |  | **1** |  | **3** |
| Delta |  | **2** |  | **6** | **8** |
| Ebonyi | **1** | **23** | **17** | **13** | **54** |
| Edo | **17** | **24** | **44** | **21** | **106** |
| Ekiti |  | **1** |  |  | **1** |
| Enugu |  | **1** | **1** | **2** | **4** |
| FCT |  | **3** | **2** | **2** | **7** |
| Gombe | **1** | **4** | **1** | **1** | **7** |
| Imo |  |  |  |  | **0** |
| Jigawa |  |  |  |  | **0** |
| Kaduna | **2** | **1** |  | **2** | **5** |
| Kano | **5** |  |  | **1** | **6** |
| Katsina |  |  |  | **2** | **2** |
| Kebbi |  |  | **1** | **1** | **2** |
| Kogi | **1** | **4** | **3** | **5** | **13** |
| Kwara |  |  |  |  | **0** |
| Lagos | **3** |  |  |  | **3** |
| Nasarawa | **6** | **3** | **4** | **4** | **17** |
| Niger |  |  |  |  | **0** |
| Ogun | **2** |  |  |  | **2** |
| Ondo | **11** | **25** | **45** | **50** | **131** |
| Osun |  | **1** |  |  | **1** |
| Oyo |  |  | **1** | **1** | **2** |
| Plateau | **8** | **9** | **10** | **5** | **32** |
| Rivers |  | **1** | **2** | **3** | **6** |
| Sokoto |  |  |  | **1** | **1** |
| Taraba | **12** | **6** | **8** | **19** | **45** |
| Yobe |  |  |  |  | **0** |
| Zamfara |  |  | **1** |  | **1** |
| **Total** | **79** | **125** | **156** | **156** | **516** |

Number of Confirmed Cases from 2017 to 2020 According to Months

|  | **December, 2016** | **January** | **February** | **March** | **April** | **May** | **June** | **July** | **August** | **September** | **October** | **November** | **December** | **Total** |
| --- | --- | --- | --- | --- | --- | --- | --- | --- | --- | --- | --- | --- | --- | --- |
| 2017 | 27 | 3 | 37 | 106 | 10 | 2 | 10 | 17 | 37 | 18 | 6 | 17 | 8 | 298 |
| 2018 |  | 67 | 193 | 45 | 21 | 9 | 14 | 24 | 24 | 17 | 39 | 27 | 48 | 528 |
| 2019 |  | 211 | 192 | 106 | 39 | 16 | 22 | 37 | 18 | 36 | 40 | 39 | 40 | 796 |
| 2020 |  | 258 | 515 | 179 | 41 | 30 | 19 | 11 | 91 | 21 |  |  |  | 1165 |
| **Total** | **27** | **539** | **937** | **436** | **111** | **57** | **65** | **89** | **170** | **92** | **85** | **83** | **96** | **2787** |

Number of Death from 2017 to 2020 According to Months

|  | **December, 2016** | **January** | **February** | **March** | **April** | **May** | **June** | **July** | **August** | **September** | **October** | **November** | **December** | **Total** |
| --- | --- | --- | --- | --- | --- | --- | --- | --- | --- | --- | --- | --- | --- | --- |
| 2017 | 16 | 0 | 18 | 26 | 1 | 0 | 1 | 4 | 5 | 3 | 1 | 4 | 0 | 79 |
| 2018 |  | 17 | 32 | 14 | 8 | 2 | 3 | 8 | 8 | 0 | 9 | 4 | 20 | 125 |
| 2019 |  | 39 | 44 | 22 | 7 | 2 | 6 | 8 | 1 | 6 | 5 | 4 | 12 | 156 |
| 2020 |  | 56 | 56 | 22 | 5 | 4 | 6 | 1 | 4 | 2 |  |  |  | 156 |
| **Total** | **16** | **112** | **150** | **84** | **21** | **8** | **16** | **21** | **18** | **11** | **15** | **12** | **32** | **516** |
